# Supplementary material for: Causal interplay between lactose intolerance and gut microbiota: a combined bidirectional Mendelian randomization and in vivo validation study
Source: Front Nutr. 2026 Jun 1;13:1803337. doi: 10.3389/fnut.2026.1803337 (PMC13265576; doi:10.3389/fnut.2026.1803337)
Supplement: Supplementary file 1 [file Data_Sheet_1.zip › supplementary materials/Forward/forest plot/ebi-a-GCST90027821.finngen_R12_E4_LACTONAS.pdf]

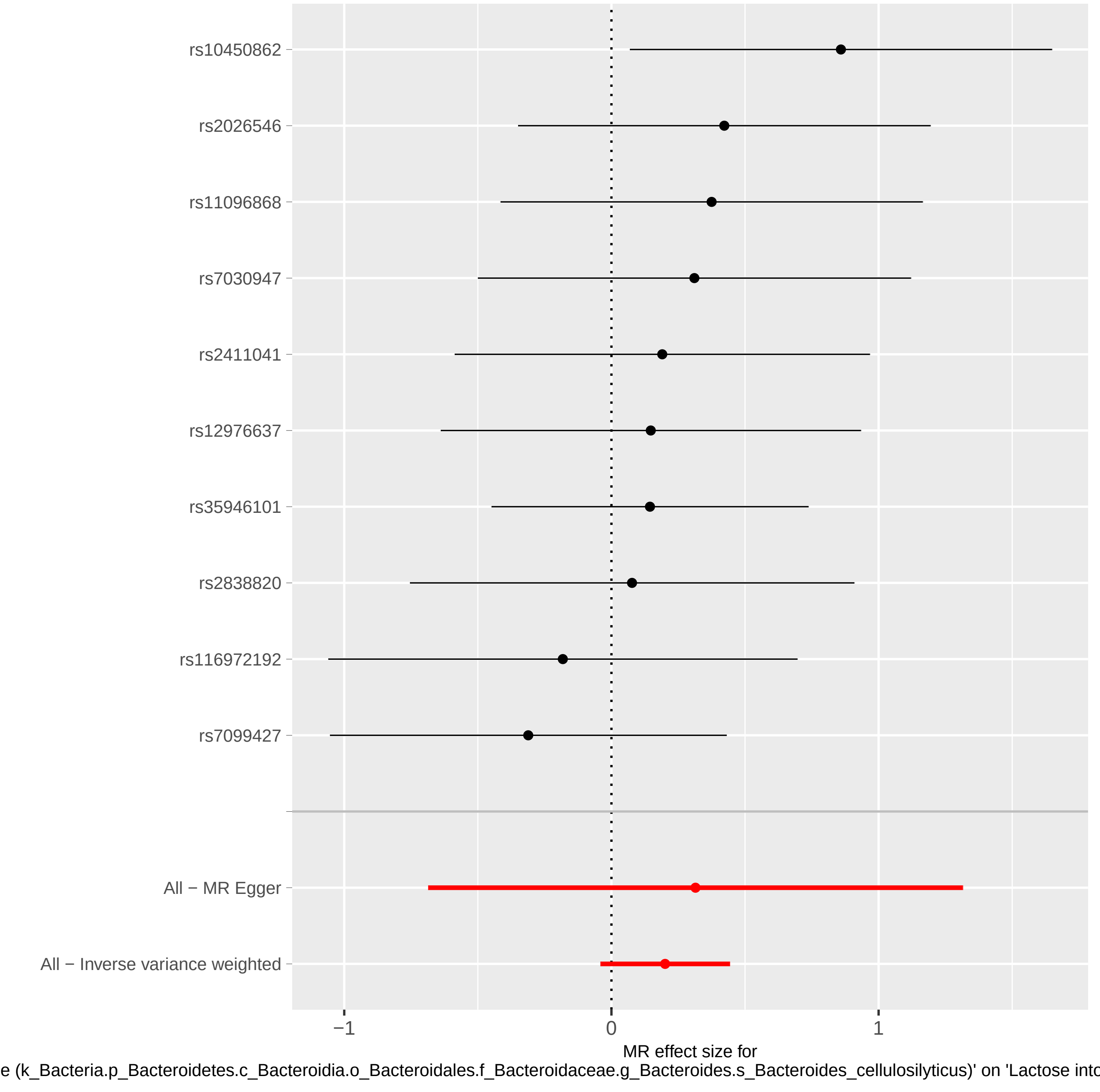
rs10450862
rs2026546
rs11096868
rs7030947
rs2411041
rs12976637
rs35946101
rs2838820
rs116972192
rs7099427
All − MR Egger
All − Inverse variance weighted
−1
0
1
MR effect size for
e (k_Bacteria.p_Bacteroidetes.c_Bacteroidia.o_Bacteroidales.f_Bacteroidaceae.g_Bacteroides.s_Bacteroides_cellulosilyticus)' on 'Lactose into
